# Effects of magnesium on the performance of sows and their piglets

**DOI:** 10.1186/2049-1891-5-39

**Published:** 2014-08-09

**Authors:** Jianjun Zang, Jingshu Chen, Ji Tian, Aina Wang, Hong Liu, Shengdi Hu, Xiangrong Che, Yongxi Ma, Junjun Wang, Chunlin Wang, Guanghua Du, Xi Ma

**Affiliations:** 1 State Key Laboratory of Animal Nutrition, Ministry of Agriculture Feed Industry Centre, China Agricultural University, Beijing 100193, China; 2 Weifang Business Vocational College, Zhucheng, Shandong 262234, China; 3 College of Animal Science and Veterinary Medicine, Shanxi Agricultural University, Taigu, Shanxi 030801, China

**Keywords:** Gilts, Magnesium, Piglets, Reproduction, Sows

## Abstract

The objective of this study was to evaluate the effects of supplemental magnesium (Mg) on the performance of gilts and parity 3 sows and their piglets. Fifty-six gilts (Trial 1) and 56 sows (Trial 2) were assigned to one of 4 treatments according to their mating weight, respectively. The treatments comprised corn-soybean meal based gestation and lactation diets (0.21% magnesium) supplemented with 0, 0.015, 0.03, or 0.045% Mg from mating until weaning. The results showed that magnesium supplementation significantly (*P* < 0.05) reduced the weaning to estrus interval in both gilts and sows. There were significant effects (*P* < 0.05) of supplemental magnesium on the total number of piglets born, born alive and weaned in sows. In late gestation and lactation, the digestibility of crude fiber (quadratic effects, *P* < 0.05), and crude protein (*P* < 0.05), were significantly influenced by magnesium in gilts and sows, respectively. There were differences among the 4 groups in terms of the apparent digestibility of dry matter and crude fiber in sows (*P* < 0.05) during both early and late gestation. The apparent digestibility of gross energy was increased for sows in late gestation (*P* < 0.05), and lactation (quadratic effects, *P* < 0.05). At farrowing and weaning, serum prolactin levels and alkaline phosphate activities linearly increased in sows as the Mg supplementation increased (*P* < 0.05). Serum Mg of sows at farrowing and serum urea nitrogen of sows at weaning was significantly influenced by Mg supplementation (*P* < 0.05). The Mg concentration in sow colostrum and the serum of their piglets were increased by supplemental magnesium (*P* < 0.05). In addition, growth hormone levels were linearly elevated (*P* < 0.05) in the serum of piglets suckling sows. Our data demonstrated that supplemental magnesium has the potential to improve the reproduction performance of sows, and the suitable supplemental dose ranged from 0.015% to 0.03%.

## Background

The reproductive performance of high producing sows has increased dramatically over the past decades [1] which may contribute to the changes in nutritional requirement of high producing sows [2]. In addition, confinement feeding of swine has resulted in the removal of nutrients and minerals from the soil to animals [3].

It is an important cofactor of several enzymes involved in protein and energy metabolism which also is a constituent of bone (NRC, 1998) [4]. It is considered one of the essential macro-minerals for swine [5]. However, researchers generally thought that it was unnecessary to supplement magnesium in diets of sows [6], since common corn-soybean meal diets can supply magnesium at levels 0.14 to 0.18% [7], which at least are 3 times more than the NRC (1998) recommendation of magnesium for sows (0.04%) [4].

The precise determination of Mg requirements of farm animals is necessary, depending on the stage of growth, performance and reproduction of the animals [8]. Magnesium supplementation improved the conception rate of sows by 11-15% and reduced the wean to service interval by 9 days. The improved conception rate may also have been influenced by Mg supplementation of boars used to service the sows [9]. However, studies on the relationship between magnesium and sows performance are scarce. The accurate effects of supplemental magnesium in sow diets on the performance are still unknown, especially the dose-effect relationship. Thus, this study was conducted to reveal the effects of magnesium supplementation in gestation and lactation diets on the performance of sows and gilts as well as their piglets.

## Materials and methods

### Ethics statement

The experimental protocol was approved by the Animal Care and Use Ethics Committee of China Agricultural University (Beijing, China).

### Experimental design and animals

Fifty-six gilts (Trial 1) and 56 sows (Trial 2) were either assigned into one of 4 treatments according to mating weight. The treatments contained corn-soybean meal based gestation and lactation diets (0.21% magnesium). The basal diets were supplemented with 0, 0.015%, 0.03%, or 0.045% magnesium from mating until weaning. Magnesium, in the form of MgSO_4_•7H_2_O (17.4% Mg) was added at the expense of corn (Table 1). Fourteen gilts (mean BW = 141 ± 5.7 kg) or sows (mean BW = 237 ± 8.8 kg) were fed each diet. All diets were fortified to meet or exceed the nutrient requirements recommended by the NRC (1998) [4]. The guidelines of the China Agricultural University Animal Care and Use Ethics Committee is referred to the Regulations of Laboratory Animal of China published in 1988 [10].

**Table 1:** **Ingredient composition and chemical analysis of the gestation and lactation diets fed to gilts and sows** (**as**-**fed basis**)

**Item**	**Gestation**	**Lactation**
Ingredients, %		
Corn	62.94	60.38
Soybean meal	16.78	20.10
Extruded soybean	-	14.00
Glucose	-	1.50
Wheat bran	16.27	-
Dicalcium phosphate	2.05	1.96
Limestone	1.24	1.21
Sodium chloride	0.32	0.45
Vitamin mix^1^	0.04	0.04
Mineral mix^2^	0.20	0.20
Choline chloride (50%)	0.16	0.16
Analyzed nutrient levels, %		
Dry matter	88.00	88.00
Crude protein	16.60	17.10
Crude fiber	4.90	5.39
Calcium	1.14	1.23
Total phosphorus	0.59	0.60
Magnesium	0.21	0.21
Calculated nutrient levels		
Metabolizable energy, kcal/kg	3,263	3,270
Lysine, %	0.56	0.98
Methionine + cystine, %	0.37	0.45
Threonine, %	0.46	0.60
Tryptophan, %	0.11	0.18

^1^Supplied per kilogram of complete diet: vitamin A, 4,000 IU; vitamin D_3_,1,000 IU; vitamin E, 75 IU; vitamin K_3_, 0.86 mg; riboflavin, 6.0 mg; pantothenic acid, 15.0 mg; niacin, 30 mg; cobalamin, 0.025 mg; biotin, 0.2 mg; folic acid, 0.6 mg; thiamine, 5.0 mg; pyridoxine, 6 mg.

^2^Supplied per kilogram of complete diet: iron, 45.0 mg; zinc, 35.0 mg; manganese, 10.0 mg; iodine, 0.2 mg; copper, 6.0 mg; selenium, 0.17 mg.

### Experimental procedures

Sows were individually housed in 2.20 m × 0.65 m gestation crates during the first 107 ± 1 d of gestation and were then moved to farrowing crates (2.2 m × 1.8 m) 7 d prior to farrowing. Gilts were offered 1.8 kg of gestation feed in 2 feedings per day from mating to d 84 of gestation and 2.5 kg of gestation feed in 2 feedings per day from d 85 of gestation until movement into lactation, while sows were offered 2 kg in 2 feedings per day from mating to d 84 of gestation and 3 kg feed per day in 2 feedings from d 85 of gestation to movement into lactation. After farrowing, gilts and sows were fed the lactation diets 3 times per day *ad libitum* and litters were standardized to 11 piglets by cross fostering within treatments during the first 48 h postpartum. Piglets were weaned at 28 d of age and had no access to creep feed. Sows and piglets were given free access to water throughout the experimental period. Ambient temperature in the gestation and farrowing rooms was maintained between 18–22°C.

The numbers and weights of individual pigs were recorded at birth and weaning. Only those pigs alive at first weighing were regarded as born alive. Defacation was observed to find out if magnesium had any effect on lowering the incidence of constipation. Blood samples were collected from ear vein of 7 gilts or 7 sows per treatment and from precaval vein of their piglets (4 males and 4 females per litter) 12 h after farrowing, also at 28-d weaning gilts and sows without fasting, and therefore the values reflect the dietary supply of nitrogen and amino acids, or the dietary imbalance of nitrogen and amino acids. Then the blood was put into heparinized tubes. Samples were centrifuged at 3,000 × g for 15 min at 4°C, and serum was stored at -20°C for assays of biochemical parameters. Gilts and sows serum urea nitrogen, reproductive hormone activities, as well as gilts, sows, and their piglets’ serum calcium, magnesium concentrations and growth hormone activities were determined. Analysis of gilts and sows serum urea nitrogen concentration and hormone activities in the collected materials was performed using enzyme-linked immunosorbent assay (ELISA) according to the instructions of the kits (R & D Systems Company, Minneapolis, MN).

Colostrum or milk samples were taken manually from the same mammary glands (first, third and fifth teat on both sides) of 7 examined sows in each group within or after 6 h postpartum, respectively, in order to analyze the level of calcium and magnesium. Calcium was determined following the methods of AOAC (2005) [11] while magnesium levels were tested using the method suggested by Miller et al. [12].

Chromic oxide (0.20%) was supplemented to each of diets as an inert marker for a period of 7 d before fecal collection to determine apparent nutrient digestibility. Fresh feces samples were collected by rectal massage on d 45 to 48 and d 95 to 98 of gestation as well as d 18–22 of lactation. Feces were immediately stored at -20°C until analysis. Samples were thawed, dried and mixed uniformly within each sow and sub-samples were finely ground through a 1 mm sieve for chemical analysis. Apparent total tract digestibility (ATTD) was calculated and the indigestible marker method using the following formula:

$$\mathrm{ATTD}\%\;=\;\left[1-\left(N_{F}/N_{D}\right)\;\times \;\left({\mathrm{Cr}}_{D}/{\mathrm{Cr}}_{F}\right)\right]\;\times \;100$$

Where N_F_ and N_D_ represent the nutrient concentration (%) in feces and diet dry matter respectively, and Cr_D_ and Cr_F_ represent the chromium concentrations (%) in diet and fecal dry matter respectively.

Samples of diets and feces were analyzed for gross energy using bomb calorimetry (Parr Instruments, Moline, IL; AOAC, 2005) [11]. All feces and experimental diets were analyzed for dry matter (AOAC method 930.15, 2000) [13], crude protein (AOAC method 990.03, 2000) [13], ether extract (Thiex et al., 2003) [14], crude fiber (AOAC method 978.10, 2000) [13], ash (AOAC method 942.05, 2000) [13], calcium (AOAC method 927.02, 2000) [13] and total phosphorous (AOAC method 965.17, 2000) [13].

### Statistical analysis

All experimental data (n = 16) were statistically evaluated by one-way ANOVA using the GLM procedure of SAS (SAS 8.01 Institute, Cary, NC). In addition, polynomial contrasts were made to determine the linear and quadratic effects of magnesium supplementation on the various parameters measured. *P* < 0.05 was considered as significantly.

## Results

### Performance

As shown in Table 2, magnesium supplementation significantly reduced the weaning to estrus interval in gilts (linear and quadratic effect *P* < 0.05). However, the total number of piglets born and born alive, the number of piglets weaned, birth weight, and weaning weight were unaffected by magnesium supplementation for gilts (*P* > 0.05). These results agreed with a previous report which revealed that supplemental magnesium did not influence the number or weight of pigs at birth or weaning in gilts [6].

**Table 2:** **Effects of supplemental magnesium on performance of gilts and their progeny** (**Exp. 1**)

**Item**	**Supplemental magnesium, %**				**SEM**^**1**^	***P *** **value**	
	**0**	**0.015**	**0.03**	**0.045**		**Linear**	**Quadratic**
Number born per litter^2^	11.2	11.7	12.0	11.6	0.48	0.46	0.51
Number of pigs born alive per litter	10.3	10.8	10.9	10.4	0.38	0.76	0.44
Average pig weight at birth, kg	1.40	1.42	1.43	1.32	0.06	0.42	0.36
Pigs weaned per litter	9.4	9.6	9.7	9.3	0.26	0.88	0.44
Average pig weight at weaning, kg	7.02	6.91	7.01	6.89	0.34	0.24	0.96
Wean to estrus interval, d	7.4	6.6	6.9	6.3	0.25	0.01	0.04
Constipation rate^3^, %	57.1	50.0	32.1	21.4	0.36	0.03	0.35
Average daily lactation feed intake of gilts, kg	4.82	4.86	4.81	4.79	0.70	0.72	0.51

^1^*SEM* = Standard error of mean.

^2^Number of litters represented per dietary treatment.

^3^Constipation rate (%) was defined as numbers of pigs on a treatment with constipation/(total number of pigs × 28d) × 100%.

In contrast, there were significant differences among the treatments in terms of sow performance (Table 3). Supplemental magnesium significantly increased the total number of piglets born, born alive, and weaned (*P* < 0.05). The increase was particularly evident for sows fed 0.015 and 0.03% magnesium (quadratic effect, *P* < 0.05). In addition, the weaning to estrus interval was shortened (quadratic effect, *P* < 0.05) for these treatments. Katalin et al. also indicated that magnesium supplementation improved reproduction performance (conception rate and litter size) and shortened the weaning to service interval of sows fed magnesium supplemented diets from farrowing to subsequent mating [15].

**Table 3:** **Effects of magnesium on performance in parity 3 sows and their piglets** (**Exp. 2**)

**Item**	**Supplemental magnesium, %**				**SEM**^**1**^	***P *** **value**	
	**0**	**0.015**	**0.03**	**0.045**		**Linear**	**Quadratic**
Number born per litter^2^	12.1	13.7	12.8	12.4	0.37	0.93	0.04
Number of pigs born alive per litter	10.4	12.5	11.4	10.8	0.35	0.91	0.01
Average pig weight at birth, kg	1.27	1.30	1.46	1.42	0.06	0.04	0.09
Pigs weaned per litter	9.21	10.64	10.0	9.43	0.21	1.00	0.01
Average pig weight at weaning, kg	7.1	7.7	7.3	6.9	0.18	0.46	0.03
Wean to estrus interval, d	7.1	6.3	6.1	6.8	0.19	0.30	0.01
Constipation rate^3^, %	64.2	42.8	35.7	21.4	0.06	0.02	0.36
Average daily lactation feed intake of sows, kg	5.12	5.13	5.17	5.10	0.62	0.56	0.82

^1^*SEM* = Standard error of mean.

^2^Number of litters represented per dietary treatment.

^3^Constipation rate (%) was defined as numbers of pigs on a treatment with constipation/(total number of pigs × 28d) × 100%.

### Apparent total tract digestibility

The effects of magnesium on the apparent total tract digestibility (ATTD) of various chemical constituents in gilts and sows were shown in Tables 4 and 5. For gilts, the ATTD of crude protein and crude fiber were quadratically (*P* < 0.05) affected by magnesium level in late gestation, and meanwhile the ATTD of crude fiber was quadratically (*P* < 0.05) affected by magnesium level in lactation (Table 4). In each case, the ATTD of gilts fed with 0.015 or 0.03% magnesium were higher than those for gilts fed with 0.0 and 0.045% magnesium. The ATTD of gross energy was linearly (*P* < 0.05) increased by supplemental magnesium in late gestation. There was no effect of magnesium level on the ATTD of any nutrient during early gestation (*P* < 0.05)

**Table 4:** **Apparent nutrient digestibility** (%)^**2 **^**of gestation or lactation diets for gilts containing various dietary levels of magnesium** (**Exp. 1**)

**Item**	**Supplemental magnesium, %**				**SEM**^**1**^	***P *** **value**	
	**0**	**0.015**	**0.03**	**0.045**		**Linear**	**Quadratic**
**Early gestation (d 45–48)**							
Dry matter	85.5	86.6	86.5	86.2	0.23	0.49	0.67
Gross energy	83.1	82.6	81.8	81.4	0.20	0.70	0.75
Crude protein	86.4	86.8	88.3	84.9	0.14	0.12	0.54
Ether extract	86.3	80.1	87.7	85.0	0.21	0.81	0.77
Crude fiber	57.3	58.8	58.2	55.5	0.13	0.41	0.47
**Late gestation (d 95–98)**							
Dry matter	79.3	82.9	81.2	78.3	1.49	0.49	0.08
Gross energy	76.8	77.4	83.4	81.5	1.67	0.03	0.07
Crude protein	77.7	84.5	80.5	79.3	1.47	0.90	0.04
Ether extract	82.9	84.7	82.6	82.5	1.35	0.57	0.68
Crude fiber	48.9	58.3	56.4	52.0	1.08	0.44	0.01
**Lactation (d 18–21)**							
Dry matter	85.3	84.3	84.7	84.2	0.88	0.44	0.71
Gross energy	82.2	84.3	83.2	83.7	1.03	0.48	0.56
Crude protein	84.0	84.5	85.5	82.9	0.85	0.59	0.21
Ether extract	85.9	85.7	86.1	86.1	1.16	0.86	0.97
Crude fiber	48.2	53.6	58.7	50.7	0.92	0.18	0.01

^1^*SEM* = Standard error of mean.

^2^Digestibility was determined using chromium oxide as marker.

**Table 5:** **Apparent nutrient digestibility** (%)^**2 **^**of gestation or lactation diets for parity 3 sows containing various dietary levels of magnesium** (**Exp. 2**)

**Item**	**Supplemental magnesium, %**				**SEM**^**1**^	***P *** **value**	
	**0**	**0.015**	**0.03**	**0.045**		**Linear**	**Quadratic**
**Early gestation (d 45–48)**							
Dry matter	80.0	86.9	85.7	85.6	0.99	0.02	0.02
Gross energy	83.5	86.3	86.2	89.4	1.53	0.83	0.08
Crude protein	83.6	86.6	87.1	86.6	1.06	0.81	0.61
Ether extract	86.4	86.5	85.8	86.0	1.20	0.71	0.31
Crude fiber	51.7	58.6	57.4	60.1	0.98	0.01	<0.01
**Late gestation (d 95–98)**							
Dry matter	78.1	83.8	83.5	83.9	1.05	0.01	0.01
Gross energy	81.5	84.1	84.0	81.1	1.24	0.01	0.04
Crude protein	83.8	84.0	85.3	83.7	0.88	0.05	0.04
Ether extract	85.9	89.1	86.2	86.4	0.92	0.71	0.93
Crude fiber	50.3	59.9	61.9	58.5	0.81	0.01	0.03
**Lactation (d 18–21)**							
Dry matter	84.6	86.1	88.2	85.2	0.60	0.41	0.08
Gross energy	77.8	82.1	82.2	75.9	0.72	0.45	0.01
Crude protein	80.9	84.9	84.8	85.5	1.03	0.02	0.03
Ether extract	74.58	79.47	84.14	82.36	0.56	0.14	0.11
Crude fiber	78.9	79.6	84.2	78.6	1.64	0.69	0.34

^1^*SEM* = Standard error of mean.

^2^Digestibility was determined using chromium oxide as marker.

For sows during early gestation, the ATTD of dry matter and crude fiber, was linearly and quadratically affected (*P* < 0.05) by magnesium supplementation (Table 5). The ATTD of sows fed the 0.015, 0.03, and 0.045% magnesium treatments were higher than those for sows fed the 0.0 magnesium treatments.

In late gestation, the ATTD of dry matter, gross energy, crude protein, and crude fiber were linearly and quadratically affected (*P* < 0.05) by magnesium supplementation. The ATTD of crude protein (linear and quadratic effects, *P* < 0.05), and gross energy (quadratic effects, *P* < 0.05) were also affected by supplemental magnesium in lactation. However, there was no significant (*P* > 0.05) difference in terms of crude fiber in lactation. Further research is needed to clarify the role of magnesium in influencing digestibility in gilts and sows.

### Serum parameters

Magnesium level had no effect on the serum levels of magnesium in gilts at either farrowing or weaning (Tables 6). However, the serum levels of magnesium in farrowing sows, was significantly affected (*P* < 0.05) by magnesium supplementation (Tables 7). Magnesium level had no effect on the serum levels of calcium in gilts or sows at farrowing or weaning (Tables 6 and 7). Serum prolactin concentrations and alkaline phosphate activities were linearly increased (*P* < 0.05) by increasing the dietary magnesium level in sows but not gilts at both farrowing and weaning (Table 7).

**Table 6:** **Effects of magnesium on serum urea nitrogen**, **calcium**, **and magnesium concentrations as well as reproductive hormone activities in gilts** (**Exp. 1**)

**Item**	**Supplemental magnesium, %**				**SEM**^**1**^	***P *** **value**	
	**0**	**0.015**	**0.03**	**0.045**		**Linear**	**Quadratic**
**Farrowing**							
Urea nitrogen, pmol/L	8,693.8	8,257.9	8,001.4	8,087.1	255.7	0.07	0.12
Estrogen, pmol/L	68.3	69.0	70.0	73.9	2.94	0.18	0.35
Progesterone, pmol/L	4,598.4	4,340.6	4,566.0	4,273.9	140.6	0.24	0.50
Prolactin, ng/L	133.8	161.4	149.9	138.7	8.82	0.94	0.10
Alkaline phosphate, U/L	8.72	9.97	9.36	9.45	0.37	0.11	0.29
Magnesium, mg/100 mL	1.83	2.00	1.85	1.89	0.05	0.89	0.10
Calcium, mg/100 mL	8.22	8.78	8.61	8.59	0.21	0.33	0.25
**Weaning (d 28)**							
Urea nitrogen, pmol/L	8,728.6	8,524.1	8,308.1	8,591.6	214.6	0.79	0.84
Estrogen, pmol/L	12.94	13.72	13.13	13.99	0.75	0.44	0.74
Progesterone, pmol/L	243.1	221.4	239.1	255.1	4.14	0.34	0.31
Prolactin, ng/L	122.9	135.5	130.3	127.8	7.64	0.78	0.59
Alkaline phosphate, U/L	13.62	13.80	14.26	14.79	0.63	0.16	0.36
Magnesium, mg/100 mL	1.36	1.44	1.34	1.42	0.09	0.94	0.99
Calcium, mg/100 mL	5.66	5.00	5.15	5.71	0.28	0.82	0.11

^1^*SEM* = Standard error of means.

**Table 7:** **Effects of magnesium on serum urea nitrogen**, **calcium and magnesium concentrations as well as reproductive hormone activities in sows** (**Exp. 2**)

**Item**	**Supplemental magnesium, %**				**SEM**^**1**^	***P *** **value**	
	**0**	**0.015**	**0.03**	**0.045**		**Linear**	**Quadratic**
**Farrowing**							
Urea nitrogen, pmol/L	8,970.2	8,695.2	8,001.4	7,890.1	102.2	0.91	0.43
Estrogen, pmol/L	83.4	90.5	85.5	78.8	5.42	0.44	0.33
Progesterone, pmol/L	9,235.8	8,000.2	8,247.7	8,365.6	107.5	0.17	0.08
Prolactin, ng/L	138.5	152.5	185.5	195.7	7.54	<0.01	<0.01
Alkaline phosphate, U/L	8.69	9.06	8.92	10.58	0.44	0.01	0.01
Magnesium, mg/100 mL	1.76	1.93	1.99	1.95	0.06	0.02	0.02
Calcium, mg/100 mL	8.63	9.07	9.99	9.02	0.38	0.52	0.70
**Weaning (d 28)**							
Urea nitrogen, pmol/L	9,423.0	8,834.4	7,557.9	8,227.9	38.4	0.01	0.01
Estrogen, pmol/L	27.2	26.3	27.3	25.2	1.38	0.39	0.63
Progesterone, pmol/L	206.3	227.2	199.9	228.0	14.4	0.56	0.82
Prolactin, ng/L	139.3	141.2	184.6	196.8	6.1	<0.01	<0.01
Alkaline phosphate, U/L	10.7	14.2	14.8	14.9	0.65	<0.01	<0.01
Magnesium, mg/100 mL	1.73	1.94	1.97	1.86	0.09	0.27	0.10
Calcium, mg/100 mL	8.48	8.62	7.51	8.58	0.42	0.67	0.52

^1^*SEM* = Standard error of mean.

### Calcium, magnesium, and growth hormone levels in colostrums, milk and piglet serum

Levels of calcium, magnesium, and growth hormone in gilt and sow milk and piglet serum were presented in Tables 8 and 9, respectively. Magnesium supplementation had no significant effect on magnesium and calcium concentration in colostrum and milk in gilt and progeny serum. Growth hormone was unaffected as well.

**Table 8:** **The effects of magnesium on calcium**, **magnesium and growth hormone levels in colostrum and milk of gilts and piglet serum** (**Exp. 1**)

**Item**	**Supplemental magnesium, %**				**SEM**^**1**^	***P *** **value**	
	**0**	**0.015**	**0.03**	**0.045**		**Linear**	**Quadratic**
**Colostrum**							
Magnesium, mg/100 mL	9.47	8.43	7.56	7.73	0.68	0.31	0.54
Calcium, mg/100 mL	85.67	82.12	69.16	83.23	8.11	0.72	0.76
**Milk**							
Magnesium, mg/100 mL	11.20	12.61	11.90	11.47	0.27	0.83	0.29
Calcium, mg/100 mL	368.50	383.69	359.10	406.37	23.56	0.65	0.41
**Piglet serum**							
Magnesium, mg/100 mL	2.23	2.37	2.22	2.68	0.29	0.91	0.76
Calcium, mg/100 mL	8.37	9.53	9.87	11.85	3.38	0.78	0.87
Growth hormone, μg/L	6.81	14.47	9.71	12.71	1.28	0.53	0.33

^1^*SEM* = Standard error of mean.

**Table 9:** **The effect of magnesium on calcium**, **magnesium and growth hormone levels in colostrum and milk of sows and piglet serum** (**Exp. 2**)

**Item**	**Supplemental magnesium, %**				**SEM**^**1**^	***P *** **value**	
	**0**	**0.015**	**0.03**	**0.045**		**Linear**	**Quadratic**
**Colostrum**							
Magnesium, mg/100 mL	7.45	7.93	8.24	9.68	0.25	0.01	0.03
Calcium, mg/100 mL	81.73	91.58	77.57	101.73	7.89	0.17	0.39
**Milk**							
Magnesium, mg/100 mL	12.61	11.07	10.32	12.13	0.51	0.60	0.21
Calcium, mg/100 mL	271.27	274.43	230.46	384.53	39.41	0.50	0.46
**Piglet serum**							
Magnesium, mg/100 mL	1.82	2.90	2.95	2.90	0.18	0.02	0.04
Calcium, mg/100 mL	8.63	8.53	8.30	10.53	0.41	0.32	0.39
Growth hormone, μg/L	9.55	10.68	8.95	10.29	1.69	0.04	0.11

^1^*SEM* = Standard error of mean.

With increasing magnesium levels in sow lactation diets, the concentration of magnesium in colostrum was increased (linear and quadratic effect, *P* < 0.05). Serum magnesium concentration in the serum of piglets suckling sows were also increased by increased levels of dietary magnesium (linear and quadratic effect, *P* < 0.05). In addition, growth hormone levels were linearly increased in nursing progeny from sows or gilts supplemented with Mg.

## Discussion

The reproductive performance of high producing sows has increased dramatically in the past decade [1] and it is possible that the nutritional requirement of high producing sows has been altered [2]. Our results indicated that the sows appeared to respond to supplemental magnesium. It is well known that body stores of minerals become increasingly depleted in high producing sows with advancing parity [16]. Therefore, it is possible that magnesium body storage declines as the sow ages, which may increase the sow’s reliance on magnesium provided in diets. Another possible explanation for the improved reproductive performance of sows supplemented with magnesium may be related to a reduced incidence of constipation. Treatments of magnesium had lower incidence of constipation than the control group. Constipation has been shown to negatively affect the reproductive performance of sows [17] and magnesium sulfate has been successfully used as a laxative to prevent constipation in gestating and lactating sows [18]. Although it is well known that the lowest dietary level of Mg was 3.5 fold higher than NRC (2012) and 5 fold higher than NRC (1998), our data demonstrated that supplemental magnesium has the potential to improve the reproduction performance of sows, and the suitable supplemental dose ranged from 0.015% to 0.03%. Further researches will reveal the actual requirements.

Magnesium was an important cofactor of several enzymes involved in protein and energy metabolism and was involved in many biochemical processes including activation of phosphates and participation in carbohydrate metabolism [15]. Where positive effects of magnesium supplementation on ATTD were observed, it is likely that the improvements were mediated by some effect of magnesium on the activity of some of these enzymes. Further investigation will be conducted to analyze what kinds of enzymes are mediated by adding magnesium. Apart from this, effect of Mg supplementation on rate of passage and water/electrolyte balance should also be considered in the following research.

Previous reports on the effects of dietary magnesium levels on serum magnesium levels in swine are inconsistent. Harmon et al. [6] reported that an increase in dietary magnesium increased serum magnesium levels in weaned pigs [6]. However, Svajgr et al. reported that supplemental magnesium had a negative influence on serum magnesium levels in growing and finishing pigs [7]. Nuoranne et al. concluded that serum magnesium is not a reliable index of body magnesium status [19]. Excess magnesium antagonizes calcium leading to a greater excretion and lower absorption of calcium [20]. Therefore, it was expected that serum calcium levels would be reduced by magnesium supplementation. However, in agreement with the results of the current study, Harmon et al. reported that serum calcium levels were not influenced by dietary magnesium level [6]. It is well known that the main function of prolactin is to induce the mammary gland to produce milk [21]. The more milk, the faster the growth of piglets [22], which might be the reason that litter weight gain was increased by magnesium supplementation in diets fed to sows. Additionally, with increasing magnesium levels in sow lactation diets, the concentration of magnesium in colostrum was increased, as well as serum magnesium concentration in the serum of piglets suckling sows. Growth hormone levels were linearly increased in nursing progeny from sows or gilts supplemented with Mg. On the other hand, our data indicated that 0.015% Mg supplementation had a higher litter gain than the control and the other treatments (0.03 or 0.045%), although there was a statistical increase in mean piglet weight at birth between treatments (0.03 or 0.045%) and the control. Therefore, this increase in serum growth hormone levels may only partially explain the increase in litter weight gain for piglets suckling sows fed supplemental magnesium, and the dose-effect relationship between the Mg supplementation and litter gain were not linear. Further researches are needed to perform to reveal the potential mechanism.

## Conclusion

In conclusion, our data indicated that increased oral administration with magnesium could reduce the weaning to estrus interval in both gilts and sows. In addition, magnesium supplementation improved other reproductive parameters of sows, but not gilts. Supplemental Mg ranging from 0.015% to 0.03% was suitable for high producing sows. The effect appeared to be age related which may be due to depleted body stores of minerals in high producing sows as they age [16]. Therefore, it is possible that as the sow ages, magnesium stores in the body decline, increasing the sow’s reliance on the diet to provide magnesium.

## Competing interests

All the authors declare that they have no competing interests in the present work.

## Authors’ contributions

Conceived and designed the experiments: JJZ XRC XM; Performed the research JJZ JSC ANW JT SDH YXM CLW GHD; Analyzed the data: JJZ JSC HL JT SDH; Wrote and edit the manuscript: JJZ JJW XM. All authors read and approved the final manuscript.
